# 

**Published:** 1884-09

**Authors:** 


CONTENTS.

| | Page. |
|---|---|
| He [illegible]th of Cities | 159 |
| Corpulence | 160 |
| Sunlight and Health | 161 |
| Prevention of Pneumonia | 162 |
| A Cheerful Home | 164 |
| Tired Mothers. | 164 |
| Learning to Swim | 165 |
| Order and Harmony | 166 |
| Anxiety of a Dying Man | 166 |
| Pneumonia | 167 |
| Care of Children | 168 |
| Health Capital | 169 |
| Facts Worth Remembering | 170 |
| A Lay Sermon | 172 |
| Scientific Notes | 173 |
| Seaweed as an Anti-Fat | 174 |
| Feeding Horses | 174 |
| Pure Water | 176 |
| How to Get Pure Water | 178 |

